# Subnational smoke-free laws in China

**DOI:** 10.18332/tid/112665

**Published:** 2019-11-05

**Authors:** Haoxiang Lin, Chun Chang, Zhao Liu, Yunting Zheng

**Affiliations:** 1 Department of Social Medicine and Health Education, School of Public Health, Peking University, Beijing, China; 2 Tobacco Medicine and Tobacco Cessation Center, China-Japan Friendship Hospital, Beijing, China

**Keywords:** China, WHO FCTC, smoke-free law

## Abstract

**INTRODUCTION:**

Implementing comprehensive smoke-free laws is an important part of tobacco control and has been promoted since China ratified the WHO Framework Convention on Tobacco Control (WHO FCTC) in 2005. This study shows the predictors of adopting subnational smoke-free laws and their alignment with Article 8 of WHO FCTC.

**METHODS:**

The legislations of 125 cities from China’s top three city grades were assessed, covering the cities that have smoke-free laws. Logistic regression is applied to evaluate the characteristics of cities that adopted a smoke-free law. We also compare each smoke-free law with the WHO FCTC Article 8 requirements.

**RESULTS:**

Provincial capital cities were more likely to adopt smoke-free laws compared with other cities. Smoke-free laws vary from comprehensive to partial bans with major exemptions. Among the 21 cities that have enacted smoke-free laws, 9 cities prohibited smoking in all indoor workplaces, indoor public places (restaurants, bars, health facilities, government buildings and schools) and public transportation. More than half of the smoke-free laws still allow designated indoor smoking rooms. Smoke-free laws that clearly ban e-cigarettes in smoke-free areas have been implemented in only two cities (Nanning and Hangzhou).

**CONCLUSIONS:**

This study shows that a number of Chinese cities have taken legislative measures to protect people from exposure to tobacco smoke. It identifies signs of progress but also areas for improvement, such as the scope of smoke-free laws, imperfect implementation of such laws, and the potential omission of e-cigarettes from the legislation.

## INTRODUCTION

Smoking kills more than a million people in mainland China every year^1^, a number predicted to grow to 2 million by 2030 and 3 million by 2050, unless effective tobacco control programs can be implemented nationwide^2^. The Chinese Government ratified the WHO Framework Convention on Tobacco Control (WHO FCTC) in 2005 and it came into force on January 2006^3^. Protection from exposure to tobacco smoke is a core component of the FCTC (Article 8)^4^, according to which countries are required to ‘*… adopt and implement in areas of existing national jurisdiction as determined by national law and actively promote at other jurisdictional levels the adoption and implementation of effective legislative, executive, administrative and/or other measures, providing for protection from exposure to tobacco smoke in indoor workplaces, public transport, indoor public places and, as appropriate, other public places’.*

As such, implementing comprehensive smoke-free laws that cover indoor workplaces, indoor public places and public transportation provides China with one of the most effective measures to counter premature death and health disparities^5^. In this direction the legislation affairs office of the State Council published a draft on National Smoke-free Regulation in 2014^5^; however, this law has not been adopted until now due to opposition at the ministerial level. Most opposition came from the State Tobacco Monopoly Administration (STMA), a ministry-level government entity that regulates and supervises the China National Tobacco Corporation (CNTC)^6^. Moreover, STMA is also a member of the steering committee of WHO FCTC implementation in China, and can therefore easily influence the process of formulating national tobacco control policy. STMA usually interferes with tobacco control legislative processes by exaggerating the economic importance of the tobacco industry^1,6,7^.

Nevertheless, it is a notable achievement that after China ratified the WHO FCTC in 2005, many cities have issued smoke-free local laws since 2008. In 2013, a comprehensive report from the China Central Party School advocated implementing a comprehensive ban on smoking in public places: *‘In addition to banning smoking in indoor workplaces, indoor public places and public transport, efforts should be made to create conditions for the progressive realization of smoking ban in other public places. Only in this way can the exposure to secondhand smoke be greatly reduced and many lives saved’*^8^. This report was significant not only for its content, but also for the fact that it represented a strong commitment by the Chinese government to take effective measures to provide protection from exposure to tobacco smoke.

As of August 2019, many cities in mainland China have adopted subnational smoke-free laws. Some laws are fully compliant with WHO FCTC Article 8 in requiring a comprehensive smoke-free environment while others still have loopholes that allow smoking in specific indoor areas.

Hence, this study evaluated the predictors of subnational smoke-free laws in China and compared each smoke-free law with WHO FCTC Article 8 requirements, with the goal of providing valuable suggestions for future smoke-free legislation and amendments at the city level.

## METHODS

### City grades and sample

Chinese cities were classified into 5 unofficial grades (Grade I being the highest and V being the lowest rated) according to GDP, per capita income, population, and number of high-level universities, transnational companies and embassies, including other indicators such as transportation situation. The Grade I cities have strong economic foundations and a large number of highly educated middle-class residents, convenient transportation, as well as considerable political resources and regional influence. Based on the above indicators, 19 cities were classified as Grade I, 36 cities as Grade II, 74 cities as Grade III, 76 cities as Grade IV, and 200 cities as Grade V, in mainland China^9^. The 129 cities in the top three grades were selected for our research, taking into consideration the influence of smoke-free laws and their benefits. After excluding 4 cities for which data were not available, 125 cities covering the main cities of China with smoke-free legislation were included in the present study.

### Data collection

We obtained most laws from the city-level congress or governmental websites, and also from the China Smoke-Free Laws Research Report^10^, and socioeconomic indices from annual provincial statistics including population, GDP, city grade, and provincial capital city. Hygiene information for each city was obtained from the Department of Planning and Information, National Health Commission of China.

### Data analysis

We used quantitative methods to analyse the data. Descriptive statistics were used to present the overall status of smoke-free laws. Categorical variables were reported as percentages. Logistic regression is applied to evaluate the characteristics of cities that adopted smoke-free laws. Table 1 shows the characteristics of the cities in this study. We also compared each smoke-free law with WHO FCTC Article 8 requirements to ban smoking in indoor workplaces, indoor public places, and public transportation^4^. For indoor public places, we focused on restaurants, bars, health facilities, government buildings and schools because there is a higher risk of exposure to secondhand smoke in these venues, which were also covered in the Global Adult Tobacco Survey^11,12^. All statistical analyses were conducted in SPSS 19.0.

**Table 1:** Variables used in this study of smoke-free laws in Chinese cities, 2018

*Variables*	*Code*
City Grade	Grade I city=1; Grade II city=2; Grade III city=3
2007 GDP	GDP per capita at city level in 2007
2015 GDP	GDP per capita at city level in 2015
2007 Population	Permanent residents for a city in 2007
2015 Population	Permanent residents for a city in 2015
Smoke-free law	If a city has adopted smoke-free law=1; otherwise=0
Provincial capital city	If a city is a provincial capital city=1; otherwise=0
Years for adopting smoke-free laws	The years from when FCTC came into force until passing a smoke-free law
Hygiene city	If a city was rated as a hygiene city by Chinese government=1; otherwise=0

## RESULTS

**Figure 1:**
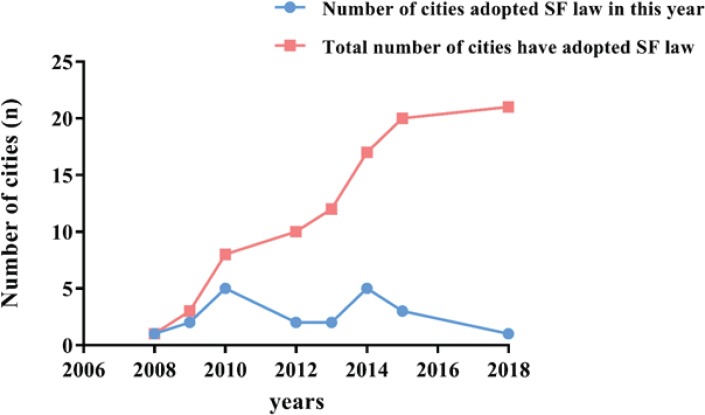
Date when 21 cities implemented smoke-free law

Of the 125 cities, more than half are Grade III cities, 30 are provincial capital cities, and 21 have FCTC-compliant smoke-free laws (Table 2). Among these 21 cities, 16 are provincial capital cities. As demonstrated in Table 3, none of the variables was statistically significant with the exception of being a provincial capital city (p<0.01). Provincial capital cities were found to be more likely to adopt smoke-free laws compared with other cities (OR=16.7). As shown in Figure 1, only a few cities adopted smoke-free laws in the first 3 years following ratification of the WHO FCTC, but the number increased from years 4 and 8 onwards.

**Table 2:** The characteristics of the 125 cities included in this study assessing smoke-free legislation in China

*Characteristics*	*Smoke-free law adopted n (%)*	*Smoke-free law not adopted n (%)*	*Total n (%)*
**City grade**			
Grade I	11 (52.4)	9 (8.7)	20 (16.0)
Grade II	5 (23.8)	31 (29.8)	36 (28.8)
Grade III	5 (23.8)	64 (61.5)	69 (55.2)
**Provincial capital city**			
Yes	16 (76.2)	14 (13.5)	30 (24.0)
No	5 (23.8)	90 (86.5)	95 (76.0)
**Hygiene city**			
Yes	16 (76.2)	72 (69.2)	88 (70.4)
No	5 (23.8)	32 (30.8)	37 (29.6)
**Total**	21 (16.8)	104 (83.2)	125 (100.0)

**Table 3:** The influence of potential variables on the existence of a smoke-free legislation

*Variables*	*B*	*SE*	*Wald*	*df*	*Sig*	*OR ( 95% CI)*
2007 per capita GDP	0.000	0.000	0.124	1	0.725	1.000 (1.000–1.000)
2015 per capita GDP	0.000	0.000	0.105	1	0.746	1.000 (1.000–1.000)
2007 population	0.000	0.004	0.006	1	0.938	1.000 (0.992–1.008)
2015 population	0.000	0.003	0.016	1	0.899	1.000 (0.994–1.007)
Provincial capital city	2.815	0.752	14.007	1	0.000	16.691 (3.822–72.891)
Hygiene city	0.091	0.713	0.016	1	0.899	1.095 (0.270–4.431)
**City grade**						
First	0.748	1.144	0.428	1	0.513	2.113 (0.224–19.901)
Second	-0.297	0.829	0.129	1	0.720	0.743 (0.146–3.771)

Table 4 presents the scope of all subnational smoke-free laws in China. Smoke-free laws vary from comprehensive bans to partial bans with major exemptions. Among the 21 cities that have enacted smoke-free laws, 9 cities have reached or are very close to reaching FCTC Article 8 requirements by prohibiting smoking in all indoor workplaces, indoor public places (restaurants, bars, health facilities, government buildings and schools) and public transportation. For indoor public places, smoke-free laws cover mostly health facilities and schools, followed by government buildings. However, the percentage of smoke-free laws that cover restaurants and bars was relatively low. For public transportation, 66.7% of smoke-free laws prohibit smoking in any vehicle used for the carriage of members of the public, and in waiting areas such as platforms.

**Table 4:** The scope of all subnational smoke-free laws in China (n=125)

*City*	*Grade*	*Indoor workplace*	*Indoor public places*					*Public transportation*
			*Restaurants*	*Bars*	*Health facilities*	*Government buildings*	*School*	
Beijing	I	√	√	√	√	√	√	√
Qingdao	I	√	√	√	√	√	√	√
Shenzhen	I	√	√	√	√	√	√	√
Anshan	III	√	√	X	√	√	√	√
Lanzhou	III	√	√	√	√	√	√	√
Nanning	II	√	√	√	√	√	√	√
Haerbin	II	√	P	√	√	√	√	√
Tianjin	I	√	P	P	√	√	√	√
Fuzhou	I	√	P	P	√	P	√	P
Hangzhou	I	√	P	P	√	√	√	√
Tangshan	III	√	√	√	√	√	√	√
Shanghai	I	√	√	√	√	√	√	√
Dalian	I	P	X	X	P	X	P	√
Shijiazhuang	II	P	X	X	P	X	P	P
Changchun	III	√	√	√	√	√	√	√
Guangzhou	I	√	P	P	√	P	√	P
Xining	III	√	P	P	√	√	√	P
Huhehaote	II	P	X	X	√	X	√	P
Yinchuan	III	P	P	P	√	P	P	P
Xian	I	√	√	√	√	√	√	√
Nanjing	I	P	X	X	P	X	P	P
√								
n (%)		16 (76.2)	10 (47.6)	10 (47.6)	18 (85.7)	14 (66.7)	17 (81.0)	14 (66.7)
P								
n (%)		5 (23.8)	7 (33.3)	6 (28.6)	3 (14.3)	3 (14.3)	4 (19.0)	7 (33.3)
X								
n (%)		0	4 (19.0)	5 (23.8)	0	4 (19.0)	0	0

√ – Smoke-free law covered area. P – Area where smoke-free law is only partially covered. X – Area where smoke-free laws are not covered.

As seen in Table 5, more than half of the smokefree laws still allow designated indoor smoking rooms, and only 8 laws clearly ban indoor smoking areas. For owners or managers of venues, all smokefree laws require ‘No Smoking’ signs at appropriate locations, and most laws require removal of any smoking-related paraphernalia from the premises and discouragement of individuals from smoking on the premises. The least represented aspects of the laws were those that required the removal of tobacco company advertisements and provision of a telephone number for the public to report violations.

**Table 5:** Existence of an indoor smoking room and responsibilities of owners or managers of venues in China

*City*	*Grade*	*Ban indoor smoking room*	*Responsibilities of owners or managers of venues*				
			*Post clear signs*	*Indicate telephone number for public to report violations*	*Remove smoking paraphernalia*	*Discourage individuals from smoking*	*Ban tobacco advertisements*
Beijing	I	√	√	√	√	√	√
Qingdao	I	√	√	√	√	√	√
Shenzhen	I	√	√	√	√	√	√
Anshan	III	X	√	√	√	√	√
Lanzhou	III	√	√	√	√	√	√
Nanning	II	X	√	X	√	√	X
Haerbin	II	X	√	√	√	√	X
Tianjin	I	X	√	√	√	√	X
Fuzhou	I	X	√	√	√	√	√
Hangzhou	I	X	√	X	√	√	√
Tangshan	III	√	√	√	√	√	X
Shanghai	I	√	√	√	√	√	X
Dalian	I	X	√	X	√	X	√
Shijiazhuang	II	X	√	X	X	√	X
Changchun	II	√	√	√	√	√	√
Guangzhou	I	X	√	√	√	√	√
Xining	III	X	√	√	X	√	X
Huhehaote	II	X	√	X	√	√	√
Yinchuan	III	X	√	X	√	√	√
Xian	I	√	√	√	√	√	X
Nanjing	I	X	√	X	√	√	√
√							
n (%)		8 (38.1)	21 (100)	14 (66.7)	19 (90.5)	20 (95.2)	13 (61.9)
X							
n (%)		13 (61.9)	0	7 (33.3)	2 (9.5)	1 (4.8)	8 (38.1)

√ – Responsibilities imposed for owners of venues by smoke-free law. X – Responsibilities are not imposed by smoke-free law.

Smoke-free laws that clearly ban the use of e-cigarettes in smoke-free areas were only recently enacted in two cities (Nanning and Hangzhou).

## DISCUSSION

This study reports on the *status quo* of smoke-free laws in China, and provides the most comprehensive picture of smoke-free laws at the city level to date. The number of cities that have enacted WHO FCTC compliant smoke-free laws has increased since the Chinese government ratified the Convention, however, the distribution is not uniform with most being provincial capital cities. One possible reason is provisional cities are the political and economic centers of the provinces, have more international communication and have both the willingness and capability to obtain financial resources for the legislation and law enforcement. Also, local authorities pay more attention to their city image and have a strong will on tobacco control. Another possibility is that the local CDC and civil societies in the developed cities have more professional and technical ability to support the local government in adopting smoke-free laws. For example, the Shanghai city Health and Family Planning Commission provided persistent and strong support during the legislation process, and international and domestic NGOs and mass media supported the legislation through community campaigns^13^. Shenzhen city amended its smoke-free law in 2014 to meet the requirements of the WHO FCTC. In addition to the support from the government and the local CDC, the Guangzhou city Tobacco Control Association and International Union against Tuberculosis and Lung Disease (Union) funded the legislative process and follow-up surveys to show the effectiveness of the law.

According to the models of Diffusion of Innovations, the innovation-decision process involves five steps: knowledge, persuasion, decision, implementation, and confirmation. During the process, people were classified as innovators, early adopters, early majority, late majority, and laggards^14,15^. The 21 cities that had enacted smoke-free laws at the time of our study were likely innovators or early adopters and may play a central role at the initial stage of promoting subnational smoke-free laws in China. Their positive evaluations about the legislation and implementation of the law may carry this action forward so that other cities also adopt such legislation.

WHO FCTC Article 8 Guidelines state that there is no safe level of exposure to tobacco smoke, that any measures, including air filtration and separate ventilation, cannot protect against exposure to tobacco smoke, and the only effective way to provide protection from exposure to tobacco smoke is to ensure a 100% smoke-free environment^16^. Studies conducted in Mexico and European countries have shown that comprehensive smoke-free laws have better compliance than partial laws and are more effective in reducing smoking behavior^17,18^.

Currently, less than half of smoke-free laws in China comprehensively ban all indoor smoking. This weakness in existing smoke-free laws presents a barrier to achieving a 100% smoke-free environment.

International studies have found that adult entertainment venues have higher rates of secondhand smoke exposure compared with other locations in the community^19^. Our study shows that the ban on smoking in restaurants and bars still needs to be strengthened, and these are the venues in which the tobacco industry has fought most strongly to prevent a smoke-free environment^20^. There has been some debate on whether comprehensive smoking bans would affect revenues of restaurants and bars. But recently, a growing number of studies in the literature have concluded that comprehensive smoke-free laws do not affect the revenues of restaurants, bars and other sectors of the hospitality industry^21,22^. A review article published by the Australia Center for Tobacco Control found that studies indicating that a comprehensive smoke-free law may have a negative impact on revenues were funded by tobacco companies. In contrast, all the well-designed studies report that such laws have no impact or even have a positive impact on sales^23^. This conclusion calls for continuous efforts with the aim of having a 100% smoke-free bars and restaurants.

Smoke-free legislation is the keystone of the WHO FCTC and MPOWER policy package^24^. However, comprehensive protection of the public from exposure to tobacco smoke depends upon many factors. Effective smoke-free laws play a critical role, and so it is important to monitor the implementation of the existing laws in order to modify and improve them^25^. Our study found the responsibilities imposed on owners or managers of venues can be improved by requiring signs with a telephone hotline for reporting violations and tobacco advertisements. The WHO FCTC Article 8 guidelines also suggest enforcement should focus on business establishments. In addition to fining the smokers, smoke-free laws should equally place responsibility for compliance on the owners or managers of the premises and clearly identify the actions they are required to take^18^.

Enforcement in many cities is facing challenges due to resource constrains, as in most cities of China, so that enforcement agencies cannot conduct regular inspections. In this sense, a reporting hotline is crucial for governments to monitor the implementation of smoke-free laws. In addition, this kind of ‘bottom up’ enforcement mechanism can mobilize societal and community participation.

Banning tobacco advertisements is part of WHO FCTC Article 13, but our results showed only some smoke-free laws mentioned banning tobacco advertisements, which can negate public health messages on the hazards of tobacco use^1^ as well as have a negative influence on smoke-free law compliance. In addition, the ultimate goal of smoke-free policy is not only to reduce exposure to secondhand smoke but also reduce smoking prevalence and improve health at the population level^21^. Therefore, banning all types of tobacco advertisements, sponsorship and promotion, especially in smoke-free places, will have additional value.

Nearly all current smoke-free laws in China fail to mention the use of e-cigarettes. As in many countries, this is a relatively new type of tobacco use in China. Unclear regulation of e-cigarette usage in smoke-free venues in current subnational smoke-free laws can become a potential loophole in the future. Research has found e-cigarettes can pose health risks; a systematic review study, of articles published from 1996 to 2015, concluded that passive exposure to e-cigarette vapour has the potential to lead to adverse health effects^26^. Other studies showed that the levels of some metals are higher in secondhand aerosols from e-cigarettes than in secondhand smoke^27^. In addition, the use of such devices in smoke-free venues may also present a law enforcement challenge. For example, people may not be able to clearly identify visual clues related to traditional cigarettes versus those of e-cigarettes (i.e. the visual vapor), and this may cause confusion and poor adherence to the existing smoke-free regulations.

Despite the challenges that tobacco control legislation and related stakeholders face, we still can identify some opportunities to strengthen tobacco control. First, the Chinese government has been committed to promoting a national smoke-free law since 2011. In 2014, the State Council drafted smoke-free national legislation for a comprehensive smoking ban. Although this law has not been adopted, endeavors that promote political will can pave the way for an effective comprehensive national smoke-free law^28^. Also, in 2018, the responsibility for the WHO FCTC lay within the Ministry of Industry and Information Technology, where the national tobacco industry resides. The responsibility for the WHO FCTC has only recently shifted to the Health Commission^29^. We believe this is a milestone for tobacco control in China that will strongly promote WHO FCTC implementation. Other noteworthy support has also come from related tobacco control partners, such as China’s CDC, the Think Tank, the Chinese Association on Tobacco Control from within China, and from international partners such as WHO, the Bill and Melinda Gates Foundation, Johns Hopkins University, the Campaign for Tobacco Free Kids, Vital Strategies, the International Tobacco Control Policy Evaluation Project (the ITC Project) and the US CDC etc. As in many countries, international engagement and domestic civil society participation has been an important component of tobacco control in China^6^.

In 2016, the Chinese government announced the Healthy China 2030 Plan. It is a new national strategy with ambitious targets including reduction of smoking prevalence from 27.7% in 2015 to 20% by 2030^30^. In order to achieve the overall objectives of Healthy China 2030 Plan, Healthy China Initiatives was launched in July 2019, and tobacco control is listed as Action 4^31^, a fully enforced smoking ban in all public places and an indoor smoking ban that will gradually cover all public venues^32,33^. Moreover, many important FCTC measures such as smoke-free legislation, along with tax increases and other legislation, can contribute to achieving the target reduction in smoking by 2030.

### Limitations

This study has some limitations. First, while all the current subnational smoke-free laws were covered, it is possible that some cities that have effective government regulations were missed because the regulations are not classified as ‘laws’. Second, our study only focused on the legal text and does not take into account the implementation of the law. Therefore, we had no opportunity to explore this topic in a real-world situation. It is expected that future studies may consider including all the smoke-free policies and also the implementation and compliance with the laws.

## CONCLUSIONS

This study provides new and potentially important information. It shows that, in the absence of a national law, more and more Chinese cities have taken legislative measures to protect people from exposure to tobacco smoke. It identified signs of successful progress but also areas for improvement, such as the scope of smoke-free laws, imperfect implementation of such laws, and the omission of e-cigarettes from this discourse. These loopholes are now identified and thus can be remedied. As smoke-free laws need continuous updating, this requires continued commitment from leadership and active participation of stakeholders and media, at all levels.
